# The Adaptive Significance of Tail‐Flagging: A Test in European Rabbits (*Oryctolagus cuniculus*)

**DOI:** 10.1002/ece3.71632

**Published:** 2025-06-21

**Authors:** Yuqian Huang, Reuben Evan Sparke, Tim Caro

**Affiliations:** ^1^ School of Biological Sciences University of Bristol Bristol UK; ^2^ Center for Population Biology University of California Davis California USA

**Keywords:** antipredator behaviour, defence, lagomorph, mammal colouration

## Abstract

Mammals are typically characterised by dull brown or grey colouration for camouflage, yet a number of species exhibit striking white underparts, including the underside of the tail, which can be facultatively displayed when the tail is raised. Nonetheless, the adaptive significance of raising a white tail by mammals is poorly understood. To investigate this, we observed 2169 escape events from wild European rabbits ( *Oryctolagus cuniculus* ), using human approaches, taxidermy predator models (fox, marten) and live buzzard attacks and tested seven hypotheses, including alarm signalling, quality advertisement and confusion effect. We further conducted phylogenetic comparative methods across Lagomorphs to examine whether the evolution of white tails is associated with ecological and social traits. We found that tail‐flagging is complex, conveying rather different information at distinct stages of predator encounters. Before escape, exposing the underside of the white tail seems to be an alarm signal to warn conspecifics. During escape, however, there was some evidence that it could serve as a quality advertisement signal to deter predator pursuit. It is also possible that in high local density populations, tail‐flagging behaviour could confuse predators. We could categorically reject vigilance advertisement, perception advertisement and group cohesion as explanations for this behaviour. The study sheds light on the evolutionary significance of conspicuous undersides in mammals and highlights the surprising complexity of signalling behaviours in predator–prey interactions.

## Graphical abstract

European rabbits use their white tails to warn others of danger and ward off predators, revealing the dual function of a single animal signalling behaviour. Using both field observations and phylogenetic analyses, we show that tail‐flagging may alarm other rabbits before escape and deter predators during escape. Alternative explanations, such as signalling alertness, awareness of predators, or maintaining group cohesion, were rejected.

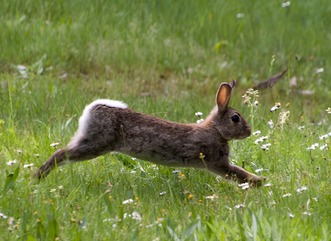

## 1 Introduction

Mammals normally exhibit dull brown or grey pelage for crypsis (Caro et al. 2017, Caro and Mallarino 2020), yet a number of species also possess highly conspicuous white undersides (e.g., Artiodactyla, Lagomorpha and Rodentia; T. Caro 2013). In species where the white underside continues to the lower aspect of the tail, tail‐flagging behaviour, often in the context of predator–prey interactions, is performed (where the tail's white underside is exposed during movement; Guthrie 1971; Caro et al. 2004).

For a biologist, understanding the adaptive significance of prey signalling in dangerous situations presents a challenge. A behaviour might be directed at predators, informing them that pursuit will be fruitless (T. M. Caro 1995), or it could be a signal to conspecifics that a predation event is imminent (Hirth and McCullough 1977); or it could be directed at both the predator and conspecifics at the same time but convey different information (Putman and Clark 2015); or it could even be directed at the predator at one stage of the predatory sequence but to conspecifics at another (Huang and Caro 2023). Additionally, it might be directed only at certain types of predators, such as coursers (T. Caro 1986; Fitzgibbon and Fanshawe 1988), because maintaining signal honesty demands predator‐specific energetic costs (T. M. Caro 1995). To some extent, different functions can be teased apart by examining the social and predator‐specific situations in which the behaviour is manifested, but findings are unlikely to be clear‐cut, more so if young, inexperienced prey who may signal inappropriately are considered.

Many lagomorphs have conspicuous undersides and tail‐flag during locomotion, and we used the European rabbit ( *Oryctolagus cuniculus* ) to explore the adaptive significance of tail‐flagging in a mammal. The European rabbit is one of the most successful invasive mammals, having established populations across Europe, Australia, the Americas and other regions (Marin‐Garcia and Llobat 2021). Very generally, this success is attributed to a combination of high reproductive output, physiological resilience and behavioural flexibility. As non‐seasonal breeders, females can produce 3–9 offspring per litter and 15–45 young annually (Rodel et al. 2008; Fernandes 2012). They are the most active during dawn and dusk, but can be seen above ground during the day, especially if their density is high or food is limited (Wilson et al. 2016). They often live in large groups with a dominant male and several females with their young in an extensive underground burrow (also known as a warren; Gunn and Morton 1995a; DiVincenti and Rehrig 2016), though smaller groups of 1–3 individuals are also common in low‐density areas (Wilson et al. 2016). Much like other lagomorphs, they have a brown‐grey coat with a white underside, including that of the tail.

One particularly notable feature of the European rabbit's behaviour is tail‐flagging: the deliberate exposure of the white tail during escape. This conspicuous visual signal may serve to warn conspecifics, deter pursuit through honest advertisement of physical quality, or momentarily confuse predators (T. Caro 2005; Stankowich 2008; Ruxton et al. 2018b), making the European rabbit a convenient model for testing the functional significance of tail‐flagging as an anti‐predator defence (DiVincenti and Rehrig 2016; Wilson et al. 2016; Marin‐Garcia and Llobat 2021).

After reviewing previous research on mammal tail‐flagging behaviours, seven hypotheses emerged: vigilance advertisement, perception advertisement, quality advertisement, alarm signalling, maintaining collective motion, group confusion and flash behaviour. Since tail‐flagging behaviour could conceivably signal different information during different stages of the escape, we considered the escape at four distinct stages from the prey's perspective—before seeing the predator (*pre‐detection*), after seeing the predator but before the escape (*preflight*), during the escape (*in flight*) and after the escape (*postflight*) – and tested each hypothesis according to which stage of predation it operates. Furthermore, we also conducted a phylogenetic comparison to investigate factors associated with the evolution of white tails across Lagomorpha. Through this multi‐pronged approach, we endeavour to provide insights into the evolutionary significance of the utility of white tails in lagomorphs and other mammals.

Vigilance advertisement (Table 1) is where a prey individual advertises its vigilance before a predator has been detected in order to deter any hidden predators from attacking (Putman and Clark 2015). Since vigilance advertisement signals often entail low energetic costs (Huang and Caro 2023), signal honesty may be maintained by the costs of individuals breaking their camouflage and increasing the likelihood of being targeted by a potential predator. Evidence for vigilance advertisement in animals is scant at present but principally derives from displaying the signal even before a predator has been detected, being associated with other vigilant postures and with a faster response if a predator initiates an attack later. For example, the tail‐wagging behaviour in white wagtails ( *Motacilla alba* ) is positively associated with scanning behaviour (Randler 2006); ground squirrels ( *Otospermophilus beecheyi* ) are more likely to flag their tails in areas where they have previously encountered rattlesnakes ( *Crotalus oreganus* ) and those that flag respond faster to simulated snake strikes (Putman and Clark 2015). Thus, if European rabbits tail‐flag to signal their vigilance to any unseen predators, then alert individuals should be more likely to flag (Table 1).

**TABLE 1:** Hypotheses and predictions for the function of tail‐flagging behaviour in European rabbits and other lagomorphs.

Hypothesis and Definition	Prediction	Explanation
**Vigilance advertisement** “*Signals used by prey that advertise its vigilance towards a potential predator even if the predator has not yet been detected*” (Huang and Caro 2023)	**Pre‐detection:** Higher probability of flagging in alert than non‐alert rabbits.	Alert rabbits are more vigilant, so are more likely to signal their vigilance to potential predators.
**Perception advertisement** “*Is directed at a stalking or ambushing predator that relies on being able to gain close proximity to the prey prior to detection; the prey signals that it has been detected prior to it coming sufficiently close to mount a successful attack*” (Ruxton et al. 2018a)	**Preflight:** The proportion of rabbits flagging is higher when approached by a fox than a marten.	The signal should be more often directed at stalking predators (fox) than coursing predators (marten).
**Quality advertisement** “*The prey signals to a coursing predator that is a particularly fleet individual, that the predator will struggle to close on, and/or a particularly strong individual, that will be difficult to subdue if the predator does succeed in closing on it*” (Ruxton et al. 2018b)	**Inflight:** Rabbits with higher running speeds are more likely to flag; and Healthy rabbits are more likely to flag than those infected with myxomatosis.	Faster rabbits are more likely to outrun the predator, so they should signal to deter pursuit; and Healthier rabbits are also more likely to outrun the predator.
**Alarm signalling** “*Warn conspecifics about detected predators*” (Fichtel and Kappeler 2002)	**Preflight and/or inflight:** The presence of offspring will increase the likelihood of flagging in adults through kin selection; Adults with more offspring in the group are more likely to flag; **Comparative analysis:** 3 White tails are more likely to evolve with larger social group sizes; and 4 White tails are more likely to evolve with larger litter sizes.	Adults with offspring should flag to warn them about potential danger; Parental fitness cost increases with increased offspring number, so adults are more willing to invest in defence for a larger litter size; Lagomorph species living in a larger group size have more related conspecifics around them; and Lagomorph species with a larger litter size may be more willing to invest in parental defence for their offspring to obtain a greater inclusive fitness and, thus, may be more likely to invest in parental defence.
**Collective motion** “*The manifestations of the locally aligned, locally synchronous and continuous movement of one or more groups of interacting individuals*” (Bode et al. 2010)	**Inflight:** Solitarily running rabbits should not tail‐flag; The proportion of rabbit tail‐flagging should be higher if they run as a whole group at the same time and all in the same direction; and **Comparative analysis:** 3 Solitary species should not evolve white tails.	Flagging should only occur when more than one rabbit runs together to maintain group cohesion; Flagging allows individuals to maintain group cohesion, which requires groups to escape at the same time and together in the same direction; and Solitary living species do not have conspecifics around them to maintain group cohesion.
**Confusion effect** “*The ability of predators to single out and track an individual prey decreases when the prey moves in a* group” (Murali et al. 2019)	**Inflight:** All rabbits from the group should escape together; Of those groups escaping rabbits, the majority of them should flag; and **Comparative analysis:** 3 Solitary species should not evolve white tails.	The confusion effect only works if rabbits escape with others in a large group; An increased proportion of flagged white tails increases the effectiveness of the confusion effect; and Solitary living species do not have conspecifics around them for group confusion effect to work.
**Flash behaviour** “*In which otherwise cryptic prey exhibit conspicuous colouration or noise when fleeing from potential predators, has been postulated to hinder location of prey once they become stationary*” (Loeffler‐Henry et al. 2018)	**Postflight:** For those rabbits that flagged inflight, they should drop their tails; Flight initiation distance (FID) and/or running distance should be longer for those rabbits that dropped their tails; and **Comparative:** 3 Species with white tails should have good camouflage at rest.	Flash behaviour only works if the prey exhibits the signal inflight and hides it again postflight; Finding the cryptic rabbit at the end is harder if it is further away from the predator; and Flash behaviour is most effective when the white tail is highly conspicuous compared to the overall body colouration.

Perception advertisement (Table 1) is another form of pursuit deterrence that operates before escape and is often directed at stalking predators (Holley 1993; T. Caro 2005; Ruxton et al. 2018a). The prey can deter a chase if it signals that it has already detected the predator and can thus start its flight at a distance far enough to escape capture. Examples include brown hares ( *Lepus europaeus* ) that show they have seen a stalking fox ( *Vulpes vulpes* ) by using an upright stance and exposing their white underside, but they do not perform this towards coursing predators such as a domestic dog (Holley 1993). Since European rabbits are predated by numerous natural predators with various hunting strategies, we would expect tail‐flagging preflight would be more likely to be exhibited if approached by a stalking predator than by a coursing predator (Table 1; Ruxton et al. 2018a).

Quality advertisement (Table 1) is a third form of pursuit deterrence, often directed at coursing predators (Bergstrom and Lachmann 2001). Here, the prey can deter further chase by signalling its ability to outdistance the predator, and the predator recognises it is a waste of energy and time to continue to pursue the prey. The honesty of quality advertisement signals is usually maintained by an energetic or probing cost of displaying the signals; thus, only fitter individuals can signal and deter pursuit (Bergstrom and Lachmann 2001). For example, energetically costly stotting behaviour displayed by Thomson's gazelles ( *Gazella thomsonii* ) during escape demonstrates their body condition and ability to outrun coursing wild dogs ( *Lycaon pictus* ; Fitzgibbon and Fanshawe 1988). In European rabbits, the whiteness of the white tail is associated with the physical condition of the individual, which could be used as a quality advertisement signal for communication with predatory owls (Penteriani et al. 2008). Therefore, if European rabbits tail‐flag to demonstrate their ability to outrun the predator, then those with a faster running speed should be more likely to flag (Table 1). In addition, myxomatosis is a widespread fatal haemorrhagic disease among European rabbits, impacting their ability to outrun the predator (Meredith 2013; Kerr et al. 2017). If flagging in flight is to demonstrate and signal an individual's quality, healthy rabbits are expected to flag more than rabbits infected with myxomatosis (Table 1).

Fourth, tail‐flagging behaviour could be an alarm signal directed at conspecifics to warn them about the presence of predators (Table 1; Fichtel and Kappeler 2002). Although the signal may be costly to the sender as it increases conspicuousness and attracts the predator's attention, the sender may still benefit through kin selection (Sherman 1977). Although auditory alarm signals have been studied most extensively (Sherman 1977; Hollen and Radford 2009; Gill and Bierema 2013; Price et al. 2015), visual signals can also be employed (Smith 1992). For example, tail‐flagging behaviour in fallow deer ( *Dama dama* ) occurs most often when the signaller is under stress or moving away from danger (i.e., a human observer), and thus the behaviour may be a signal of alarm (Alvarez et al. 1976). If tail‐flagging in European rabbits is to warn conspecifics about imminent danger from predators, then the presence of their offspring should increase their likelihood of flagging (Table 1). If the white tails had evolved for alarm signalling across many species of lagomorphs, then we would expect the evolution of white tails to be associated with a larger social group size since this maximises the impact of the warning signal (Table 1).

As solitary individuals are more likely to be targeted by predators (Ioannou et al. 2019), it might be in an individual's interest to maintain group cohesion during escape (Ioannou, Guttal, and Couzin 2012; Herbert‐Read et al. 2015; Cuthill 2019; Negro et al. 2020). By staying together, individuals should benefit from reduced attack risk via dilution, confusion, or selfish‐herd effects (Hamilton 1971; Foster and Treherne 1981; Hogan et al. 2016). Conspicuous signals could also be used in flight to facilitate collective motion (Table 1), helping animals escape together in a group (Bode et al. 2010; Ward et al. 2018; Negro et al. 2020). Conspicuous colour signals used to maintain group cohesion while moving have been suggested for many species, ranging from birds to fish to mammals (de L. Brooke 1998; Negro et al. 2020; Yu et al. 2022). If European rabbits tail‐flag to maintain collective motion, then solitary rabbits should not flag in flight, and solitary lagomorph species should not have white tails (Table 1).

Alternatively, tail‐flagging might prevent a predator from tracking an individual's flight path if group members tail‐flag together (Table 1) and thus lower the attack‐to‐kill ratio with increasing prey group size (Krakauer 1995; Ioannou et al. 2008; Schradin 2019). It has been suggested, for example, that white patches on the plumage of wading birds could be involved in group confusion when they fly in flocks (de L. Brooke 1998). Therefore, if European rabbits tail‐flag to induce a group confusion effect, we would expect them to escape together in large group sizes and all flag in flight when approached by a predator (Table 1).

Finally, flagging the tail and dropping it at the end of the flight could work together to achieve flash behaviour (Table 1). If the prey displays a conspicuous signal during the escape, a predator could be misled into anticipating the prey as always being conspicuous in appearance and, therefore, fail to find the cryptic prey when it returns to being stationary again (Loeffler‐Henry et al. 2018). Examples of flash displays are taxonomically widespread (Cott 1940; Loeffler‐Henry et al. 2019) and include the brightly coloured hindwings of many otherwise cryptic Orthoptera and Lepidoptera species (e.g., *Oedipoda caerulescens*) and the conspicuous rump and underwing coverts of many bird species (Cott 1940; Edmunds 1974; Loeffler‐Henry et al. 2019, 2021). If European rabbits tail‐flag in flight and drop their tails postflight for flash behaviour, then white tails are more likely to evolve in species with good overall body camouflage at rest (Table 1). A more conspicuous white tail compared to the rest of the body would therefore attract more attention and make the prey harder to find once the tail is hidden again (Sherratt and Loeffler‐Henry 2022). Furthermore, if European rabbits tail‐flag in flight and drop their tails postflight for flash behaviour, then they should do this at greater distances away from the predator because it will be more difficult to find rabbits at a distance (Loeffler‐Henry et al. 2021).

## 2 Methods

### 2.1 Fieldwork

#### 2.1.1 Study Area and Population

The study was conducted from April to November 2023 at eight field sites located in rural areas around Bristol, UK (51°45′45″ N, 2°58′79″ W). All sites were open dry grassland and arable margins with domestic horses and sheep at some sites. During the study, the vegetation remained consistently short across sites—either mown or grazed by livestock—exposing the pale‐brown soil underneath. This maintained visual similarity with the rabbits' natural background. Natural predators such as foxes and buzzards ( *Buteo buteo* ) were present, but no martens or other large mustelids were observed in the field sites, and no dogs were allowed in them.

Each field site contained, on average, 58.8 ± 16.9 individuals (maximum = 150, minimum = 20), although these are likely to be undercounts with other individuals below ground; over 450 different European rabbits were recorded as living in these fields during initial counts before the study began. A total of 2169 instances of European rabbits escaping were recorded. Owing to natural predation and the spread of myxomatosis, the rabbit population declined in the late summer, and we abandoned three of the eight sites in the second half of the study.

#### 2.1.2 Behavioural Data

**FIGURE 1:**
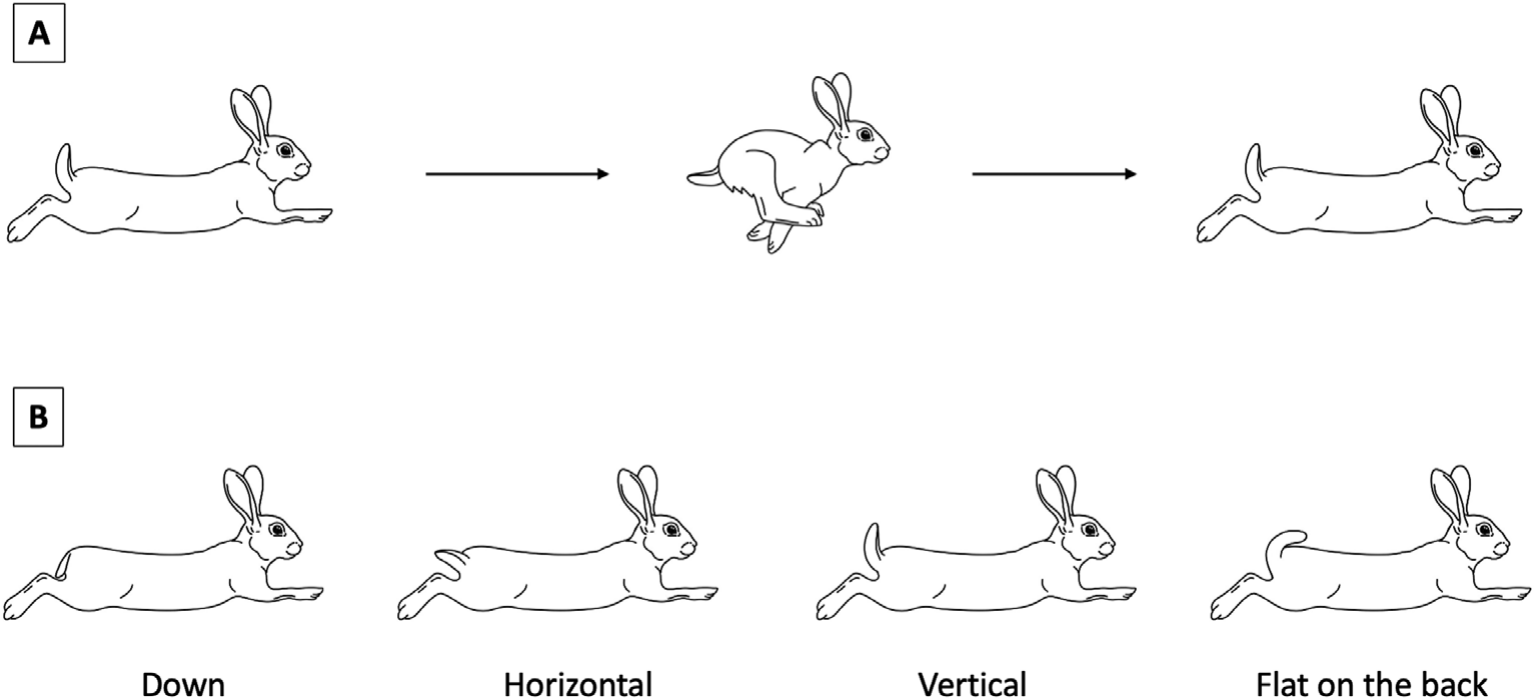
Illustrations of tail‐flagging behaviour in flight. (A) Up‐and‐down movement of the tail in flight caused by the running gesture in some lagomorphs. (B) Four tail positions during flight, depending on the angle of flagging. Tail down and horizontal were grouped as non‐flagging, whereas vertical and flat on the back flagging were grouped as flagging.

Tail‐flagging behaviour, defined as a rabbit raising and exposing the white ventral side of the tail, was separated into flagging before seeing the predator (*pre‐detection*), before escape (*preflight*), during escape (*inflight*) and after the escape (*postflight*) if the rabbit stayed outside of safety. “Safety” was defined as either a bush or a burrow entrance that occluded further observation. Tail angles were hard to classify objectively because running posture changed the tail angle (Figure 1A), but tail position *inflight* was initially categorised into down, horizontal, vertical and flat on the back (Figure 1B). Subsequently, however, flagging *inflight* was grouped into either non‐flagging (down and horizontal) or flagging (vertical and flat on the back) to reduce subjectivity. Either European rabbits almost always flagged throughout the escape or did not flag at all during escape, and thus the likelihood of flagging inflight was not influenced merely by running distance. Furthermore, tail‐flagging inflight was not an unavoidable biomechanical consequence of running in rabbits (i.e., rabbits can run fast and far without a flagged tail). To ensure this simplification did not bias our results, we repeated all analyses using the original four‐position categories (down, horizontal, vertical, flat on back) and found that the statistical outcomes remained unchanged. This supports the validity of our binary classification (flagging vs. non‐flagging) for inflight tail position.

European rabbits were classified as very young, juvenile, or adult based on estimated body size and coat colour. Very young rabbits had a yellow‐brown coat and were very small in size, approximately aged around 1–2 months; juveniles had a light grey‐brown coat and were larger, but were still slim and aged around 2–4 months; adults had a grey coat and were fully grown, aged over 4 months. We defined a “group” of rabbits if the distance between any two individuals was within 10 m of each other (Gunn and Morton 1995b; DiVincenti and Rehrig 2016). The distance between individuals was estimated visually by observers at the start of each trial, and then subsequently confirmed and corrected where necessary using a ranger finder (AOFAR HX‐800I). If there were any juvenile or very young rabbits within 10 m of an adult, then we assumed the adult had its own youngster around it. When individuals in a group escaped together at the same time, the running group size was also recorded. All other distances were also measured using a range finder to the nearest metre.

Myxomatosis was categorised based on visible signs following established clinical descriptions (Ross and Tittensor 1985; Ross et al. 1989). Individuals were classified as healthy (no signs of disease), slight (swelling on the eyelids and face, but both eyes were still open) and severe (significant swelling on the eyelids and face that stopped one or both eyes from opening).

Rabbit behaviours were recorded before the escape (Gunn and Morton 1995a; Held et al. 2001). Feeding (rabbit is crouching down and actively eating), grooming (rabbit is cleaning its body), social behaviour (rabbit is interacting with another individual(s), running, chasing, or jumping) and resting behaviours (rabbit is lying down on the ground, either with all four legs tucked underneath the body or lying on the side of the body, ears may be down on the head) were all classified as non‐alert behaviours. Alert behaviour was when the rabbit paused all other activities, its ears pointed upwards and it could stand up on its hind legs and look ahead towards any potential threat.

Finally, ground and aerial cover for the European rabbits were estimated as independent variables in terms of the difficulty of making a successful attack by a terrestrial or aerial predator on a scale of 0–5. For ground cover, we assessed bare ground with grass height < 2 cm as 0, short grassland with average grass or shrub height 2–5 cm as 1, 6–10 cm as 2, 10–15 cm as 3, 15–30 cm as 4, and 5 if the rabbit was in very dense scrub or a burrow and could not be seen by the observer from the side. Similarly, for aerial cover, we took no cover as 0, slight cover (usually between 6 and 10 m away from a hedgerow or a tree branch) as 1, some cover (usually between 1 and 5 m away from a hedgerow or a tree branch) as 2, directly underneath a hedgerow or on the edge of a tree branch as 3, directly underneath a big tree branch as 4, and 5 if the rabbit was in complete cover and could not be seen from above (e.g., directly underneath the middle of a hedgerow or a tree, or inside a burrow). Most trials involved rabbits in low‐safety environments. For ground cover, the majority had a safety score of 0 (*N* = 836), indicating bare or very sparse grass. Fewer trials had intermediate scores with short grasses (*N* = 195 for score 1, *N* = 147 for score 2), with only a small number of rabbits found in the higher safety categories (*N* = 74, 26 and 15 for scores 3–5, respectively). For aerial cover, most trials were scored as 1, meaning rabbits were in areas with only slight overhead cover, and very few rabbits were in areas offering complete aerial concealment (*N* = 76, 935, 164, 101, 12 and 5 for scores 0–5, respectively). Overall, rabbits were most often exposed or only lightly sheltered from both ground and aerial predators.

#### 2.1.3 Human Trials

**FIGURE 2:**
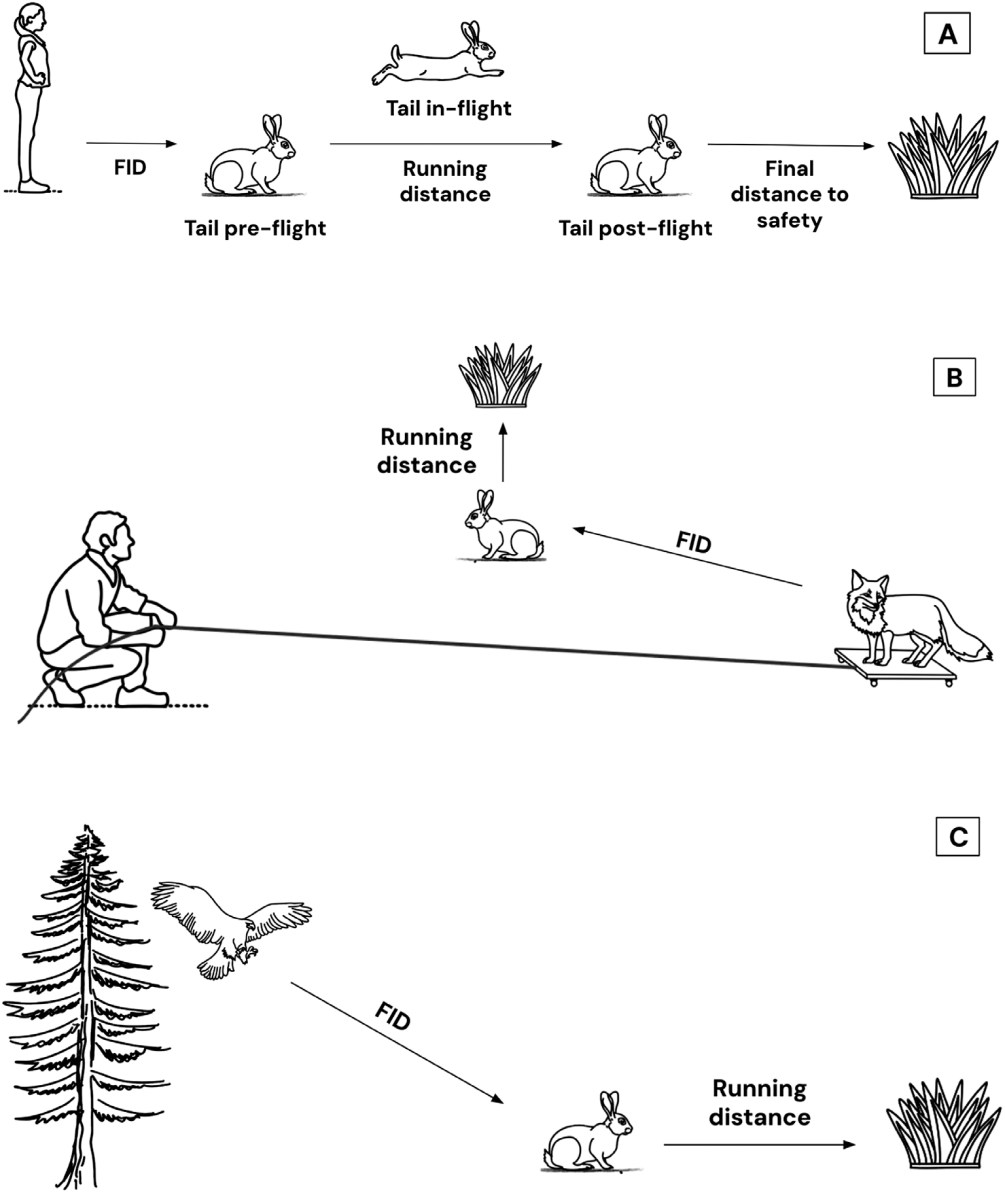
Experimental set‐up for the (A) human, (B) trolley and (C) buzzard approaches. The three tail positions (preflight, inflight, postflight) in the trolley and buzzard approaches were recorded in the same way as for the human trials. FID, flight initiation distance.

To investigate tail‐flagging behaviour, we recorded the behaviours of wild European rabbits using human participants as potential predators (Stankowich and Coss 2007). When observers approached a rabbit in the field from far away, two people first noted its tail position before seeing the observer (*pre‐detection*), its distance to the nearest place it would achieve safety using a rangefinder and the ground and aerial cover around it using binoculars (Figure 2A). As rabbits have poor visual acuity (Levick 1967; Hughes 1971) and the average observational distance was 50 m away from the focal rabbit, human observers do not generally pose an alert to the rabbits during this stage. Next, we walked towards a randomly chosen focal rabbit but stopped the approach once the rabbit started escaping and recorded the flight initiation distance (FID), running time and tail position preflight and inflight. Running distance was later measured by the observer standing in the initial position where the rabbit was before the escape and measuring the distance to the rabbit's finishing location. It thus represents the minimum distance run. If the rabbit stayed outside the burrow at the end of the flight, then its tail position postflight, the final distance to nearest safety and final ground and aerial cover around the position where the rabbit had stopped were also recorded.

#### 2.1.4 Taxidermy Animals and Trolley Trials

**FIGURE 3:**
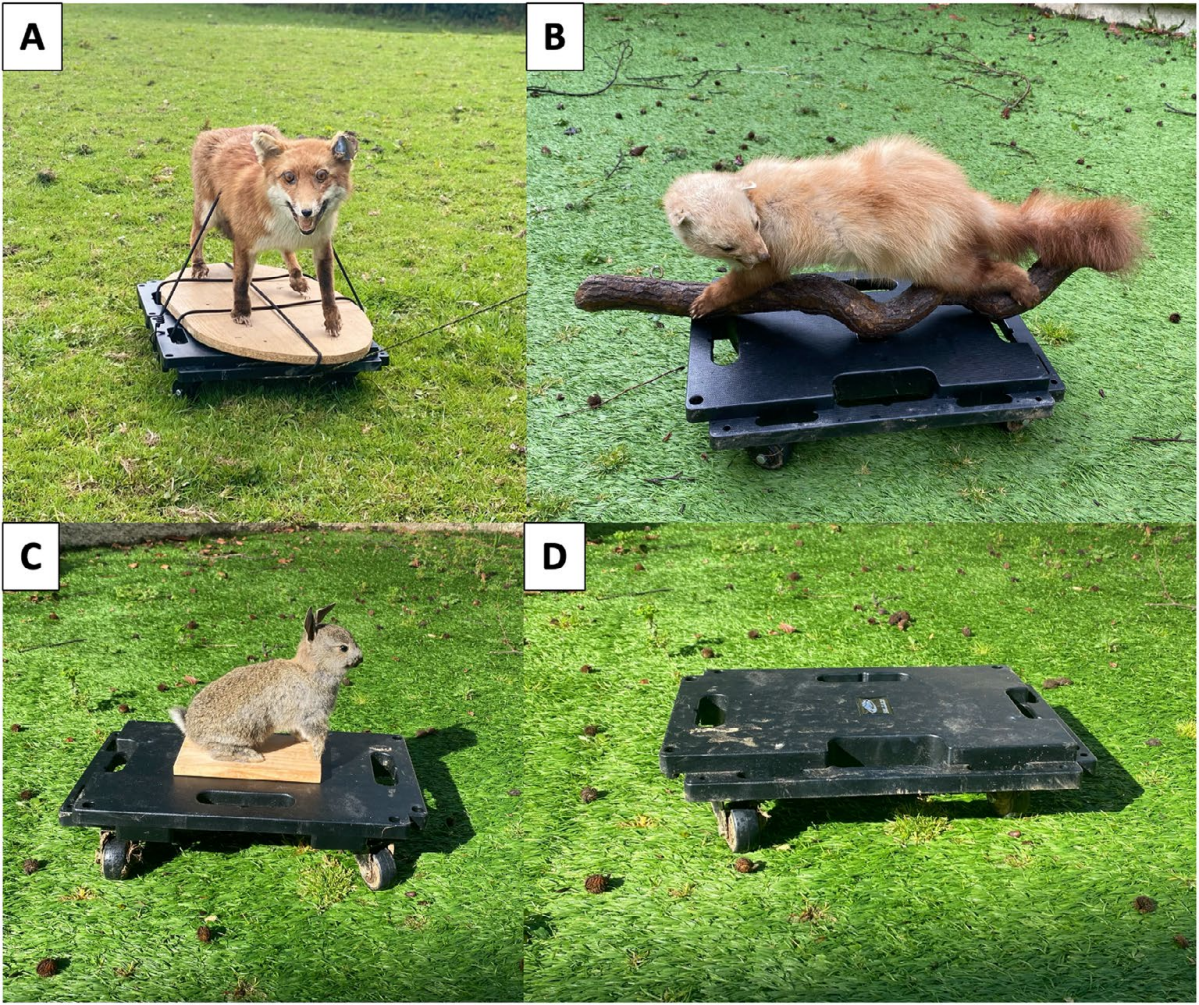
Taxidermy models and trollies used in the field. (A) Fox ( *Vulpes vulpes* ), (B) beech marten (*Marten foina*), (C) European rabbit ( *Oryctolagus cuniculus* ) and (D) empty trolley.

A stuffed red fox ( *V. vulpes* ), a stuffed beech marten (*Marten foina*) that has some similarity to a ferret ( *Mustela furo* ) and a stuffed European rabbit were used in this study (Figure 3). Foxes are stalking predators that rely on getting close to the prey before mounting successful attacks (Holley 1993). In contrast, martens use various hunting skills, including coursing, stalking and jumping from trees (Spencer and Zielinski 1983). The stuffed rabbit was used as a control for the predators. Since the stuffed rabbit had a flagged white tail, it remains unclear what signal it would convey and may alter the behaviour of the wild European rabbits. Thus, an empty trolley was also used as an additional control. Each treatment involved a single taxidermy model. Although this can be considered pseudoreplication (Wiley 2003), it reduces additional confounding factors (e.g., predator stance or size) and reflects the practical and ethical challenges of sourcing lifelike taxidermy specimens. With this in mind, we cannot exclude the possibility that the responses recorded were specific to the particular models used.

Taxidermy models were attached to a flat 55 × 40 cm trolley using bungee cords (Figure 3A), and a 110 m parachute rope was attached to the trolley in order to pull the trolley slowly at approximately 2 m/s (Figure 2B). All trollies and their models were covered with camouflaged fabric and were left in the field for at least 30 min before the start of each trial. All recordings were made of European rabbits undisturbed by human observers (i.e., they remained outside the burrow and continued with their previous activities when the human observers approached to pick up the end of the rope). Due to a lack of suitable ground, only two larger field sites were used for the trolley treatments. These trolley treatments were rotated across different areas of these two fields such that the rabbits from the same area of the field were not exposed to the same treatment more than once per week.

Before observers approached to pick up the end of the rope in each trial, the rabbit's preflight tail position was recorded at a far distance to avoid alerting the European rabbits. When the trolley was pulled, the taxidermy animals appeared from underneath the camouflaged fabric, and the trolley was stopped from moving once the rabbit started its flight. The rabbit's initial position was noted, and the initial distance between the rabbit and the trolley was measured at the end of the flight using the camouflage fabric left at the trolley's initial position. All other measurements were made in the same way as in the human trials (Figure 2B).

#### 2.1.5 Buzzard Attacks

Observations of buzzard attacks were opportunistic, recorded when buzzards were naturally present and actively hunting. There were four buzzards present in one of the field sites, and eight different attacks involving a total of 27 European rabbits were observed (Figure 2C). Once a buzzard was spotted circling above the field, observers positioned themselves on a low hill within the site to maximise visibility across the surrounding grassland. All observations of buzzard attacks took place in an open grassland habitat with shrub coverage exclusively around the edges of the field, allowing unobstructed views across much of the field. In all trials, human observers were at least 100 m away from the potential rabbit(s) approached by the buzzard, so it was assumed that rabbits were undisturbed by them. Observations were conducted using binoculars by the observers, and any events where visual obstruction occurred were excluded from the dataset. All measurements were made in the same way as the human trial, except for the FID, which was measured as the estimated distance between the buzzard and the rabbit. A buzzard was always hovering over the treelines in the field, so the location of the buzzard at the point of attack was measured using the nearest tree branch that was of similar height compared to the buzzard in the air. The observer then walked to the initial location of the rabbit and measured the distance from this location on the ground to that tree branch to estimate FID.

### 2.2 Statistical Analysis

All statistical analyses were conducted in R 4.2.3 (R Core Team 2020) using the Rstudio interface (Rstudio Team 2020). All residues of continuous data were normalised using the bestNormalize package (Peterson 2021), and variance homogeneity was checked using Bartlett's test. To evaluate the relevant variables for each hypothesis, we constructed generalised linear mixed‐effects models (GLMMs) using the ‘glmer’ function in the ‘lme4’ package (Bates et al. 2015). The tail‐flagging position (yes/no) was modelled as a binomial function of the relevant predictors for each hypothesis. Overall, a total of six predatory treatments were included—empty trolley (*N* = 58), stuffed rabbit (*N =* 43), stuffed marten (*N* = 77), stuffed fox (*N* = 90), live buzzard (*N* = 27) and human approaches (*N* = 1873). We compared the running speed, distance, time and FID between each treatment in separate linear mixed‐effects models (LMMs) using the “lmer” function in the “lme4” package (Bates et al. 2015) followed by Tukey *post hoc* pairwise comparisons using the “emmeans” function (Lenth et al. 2024). All variance homogeneity was checked using Bartlett's test. To account for repeated measures for individuals that were in the same group, we included Group ID as a random effect in all mixed‐effects models. We also included the location of the field sites, ground cover, aerial cover and initial distance to safety as random effects to account for any local adaptation. All residuals were checked using the Shapiro–Wilk normality test and normalised using the bestNormalize package (Peterson 2021).

### 2.3 Ethical Note

This research adheres to the ASAB/ABS Guidelines for the use of animals in research. The procedures presented here were approved by the University of Bristol Animal Welfare Committee (reference code UIN‐23‐039).

### 2.4 Comparative Analyses

#### 2.4.1 Data Collection

Information concerning colouration and ecological variables of 91 out of 93 species of Lagomorpha were collated from the Handbook of the Mammals of the World: Lagomorphs and Rodents (Wilson et al. 2016) and the IUCN Red List of Threatened Species.

#### 2.4.2 Colouration Variables

Body and tail colouration of adults of each species were scrutinised for any white patches of fur. We recorded the colouration of tails as ‘yes’ if the tail has a white underside and ‘no’ if the colouration is not white or if the species does not have a tail. We also examined the degree of conspicuousness for the body colouration – “0” if the overall body colouration is all dark, “1” if there were minor white patches on the body (e.g., faint white lines around the face or the ears) and “2” if there were prominent white patches on the body (e.g., white flank or white stripes across the body).

#### 2.4.3 Ecological Variables

We took absolute tail length and litter size for each species from the midpoint between maximum and minimum values. We classified the preferred habitat for each species into “Closed”, “Open”, or “Both”. Closed habitat included all types of forest and shrubland, and open habitat included all types of savanna, grassland, rocky areas and deserts across the world. Finally, we categorised species according to aspects of their social group size. Due to a lack of specific group size data, we recorded sociality as ‘1’ for solitary species, ‘2’ for groups of 2–5 and ‘3’ for groups of 6–25. These categorisations were based on the data provided in the Handbook of Mammals of the World: Lagomorphs and Rodents (Wilson et al. 2016).

#### 2.4.4 Phylogenetic Comparisons

A phylogenetic regression analysis was performed using the generalised linear mixed‐effect models in the ‘MCMCglmm’ package (Hadfield 2010) in R 4.2.1 (R Core Team 2020) using the Rstudio interface (RStudio Team 2020). For each regression analysis, we only included species for which we had information on both the tail colouration and the relevant colouration or ecological variables. Thus, the total number of species used varied between models.

All analyses were conducted as logistic regressions in a Bayesian phylogenetic model using the “MCMCglmm” package in R. The random effect included the current accepted complete node‐dated Lagomorph phylogeny from VertLife (Upham et al. 2019), and 100 tree topologies were randomly selected before the running of each model (see Howell and Caro 2024). As per Hadfield's (2010) methodology, the phylogenetic variance in all models was set to an improper prior (*V* = 10–10, *v* = −1), and the residual variance was fixed at 1. Before every model, a dummy run was performed on an arbitrary tree to determine the starting point. These dummy runs ran for 11,000 iterations, with a burn‐in of 1000 and samples were drawn every 10 iterations. The MCMCglmm models were run for 11,000 iterations, with a 1000 burn‐in period and samples drawn every 1000 iterations. Thus, a sample of 10 iterations was obtained from each tree, providing a total posterior sample of 1000. All effective sample sizes were larger than 500, and autocorrelations were checked visually by plotting the posterior distribution of MME solutions (Sol) to make sure the distribution of samples was stationary. Since species with missing data for the tested variables were trimmed from both the dataset and phylogeny prior to each model's construction, each variable was tested in a separate model as an explanatory variable for the evolution of white tails to maximise analysis power. The phylogenetic tree was plotted using the ‘ggtree’ function from the ‘ggtree’ package (Yu et al. 2017).

## 3 Results

### 3.1 General Results

A total of 2169 escape events were recorded. Before the escape, 31.8% of European rabbits were found in groups of more than one individual. During the escape, over 87.9% of the rabbits fled alone, 7.9% fled in pairs and only 4% fled in groups of three or more rabbits together. There were seven groups of rabbits found in a field consisting of 10 or more rabbits, but all escaped in group sizes of four or fewer, leaving the rest of their group in the field.

**FIGURE 4:**
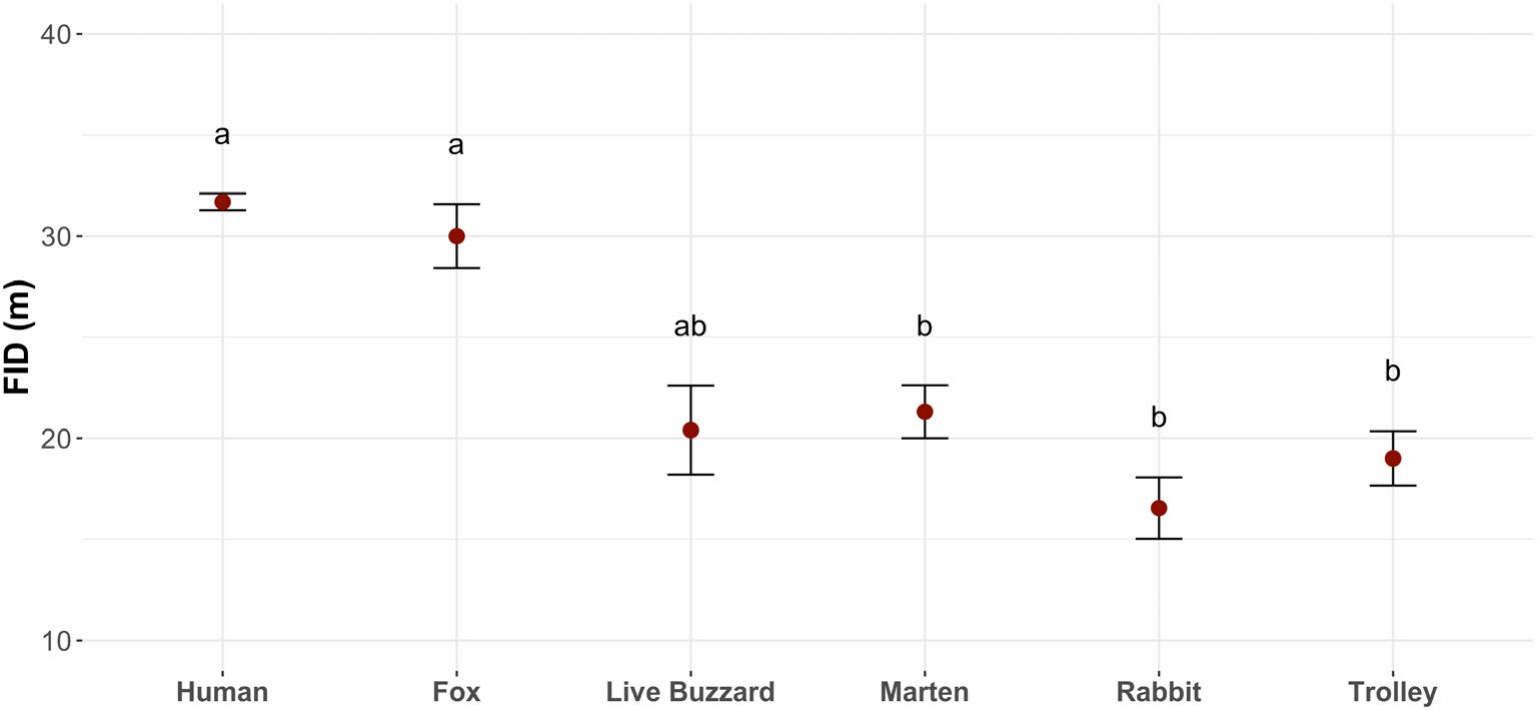
Rabbit's flight initiation distance (FID) under different treatments. The red dots represent the mean and the vertical lines delimit the standard error. Sample sizes for different treatments are indicated. Data with the same lowercase letters are not significantly different (Kenward‐roger method, *p* > 0.05).

European rabbits escaped with a higher FID when they were approached by a human (LMM: *β* = 0.932, SE = 0.199, *p* < 0.001), fox (LMM: *β* = 0.973, SE = 0.232, *p* < 0.001) and marginally for live buzzard (LMM: *β* = 1.117, SE = 0.403, *p* = 0.06) compared to a stuffed rabbit, but there was no difference compared to the marten or empty trolley (LMM: *p* > 0.1 for both treatments; Figure 4). These results indicate that wild European rabbits treated humans and foxes in the same way and treated these differently from controls, but that this was not the case for martens. As martens are not commonly seen in the southern part of the UK (Bright and Smithson 2001), this could perhaps be the reason why rabbits did not treat the stuffed marten as a potential predator.

In general, European rabbits may need to signal more if they are at greater risk of danger and thus should tail‐flag more if they are in a less covered area and hence a more dangerous environment. For *preflight*, rabbits were more likely to tail‐flag in less covered ground environments (GLMM: *β* = −0.458, SE = 0.191, *p* = 0.016), but were indifferent to aerial cover (GLMM: *β* = 0.101, SE = 0.077, *p* = 0.19). *During the escape*, however, rabbits were more likely to tail‐flag in both less covered ground and aerial environments (GLMM: *β* = −0.215, SE = 0.0870, *p* = 0.013 for ground cover; *β* = −0.400, SE = 0.0581, *p* < 0.001 for aerial cover). Finally, there was no association between tail‐flagging *postflight* and either ground or aerial cover (GLMM: *β* = −0.075, SE = 0.139, *p* = 0.59 for ground cover; *β* = −0.0640, SE = 0.0976, *p* = 0.51 for aerial cover). Furthermore, rabbits were more likely to flag during flight if they were at an intermediate distance away from the nearest safety (i.e., a burrow or a bush; GLMM: *β* = −0.242, SE = 0.060, *p* < 0.001 for the quadratic term of the distance to nearest refuges), but this association was not seen for flagging preflight or postflight.

**FIGURE 5:**
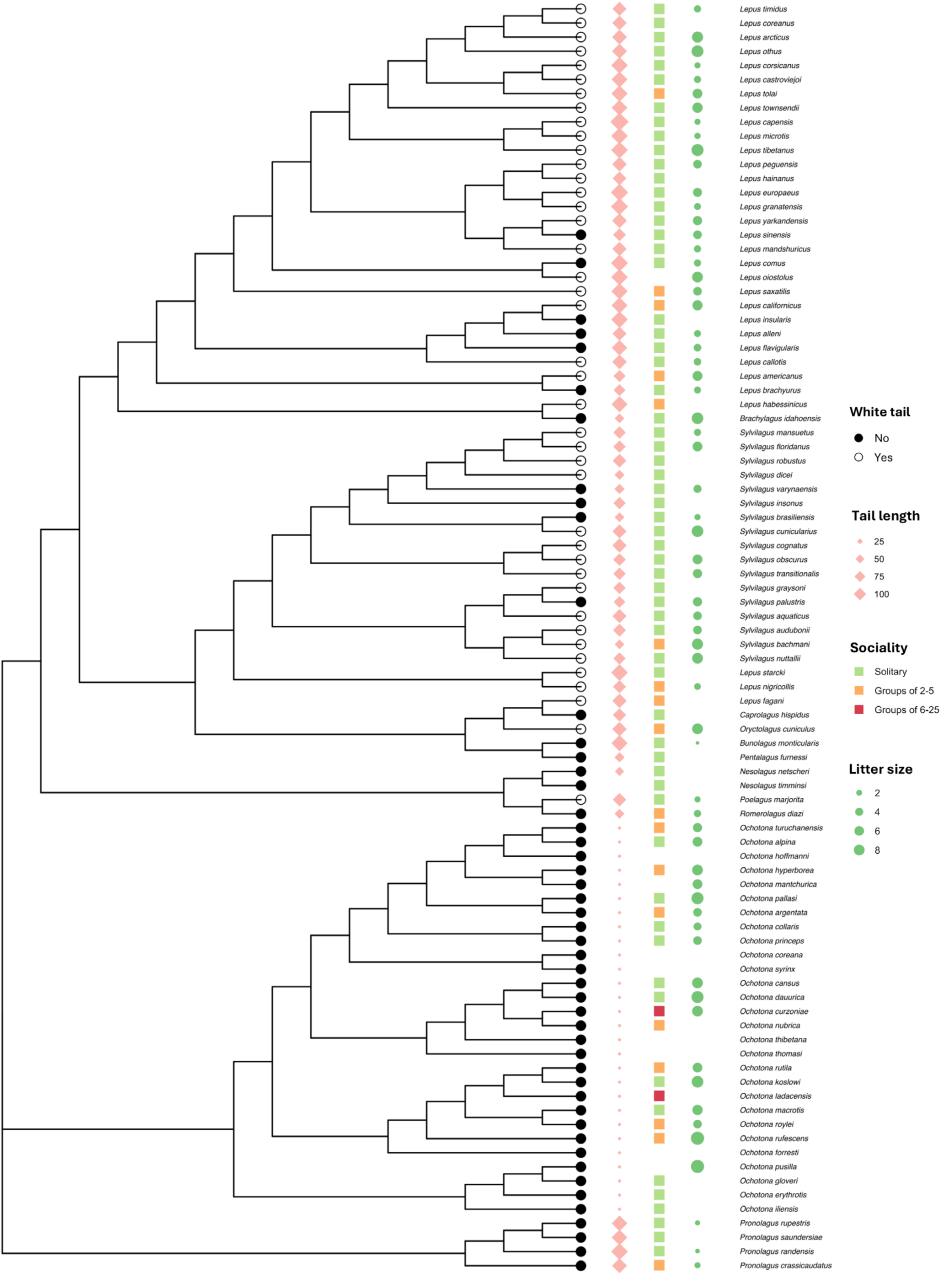
Phylogeny of lagomorph species showing the evolution of a white tail with social group size and litter size. Branch tip showing white tail (white = Yes, black = No). The size of the pink diamond represents the absolute tail length (in mm), the colour of the square represents social group size (green = Solitary, orange = Groups of 2–5, red = Groups of 6–25), and the size of the green dot represents litter size next to the species names. Blank cells are due to the absence of data.

**FIGURE 6:**
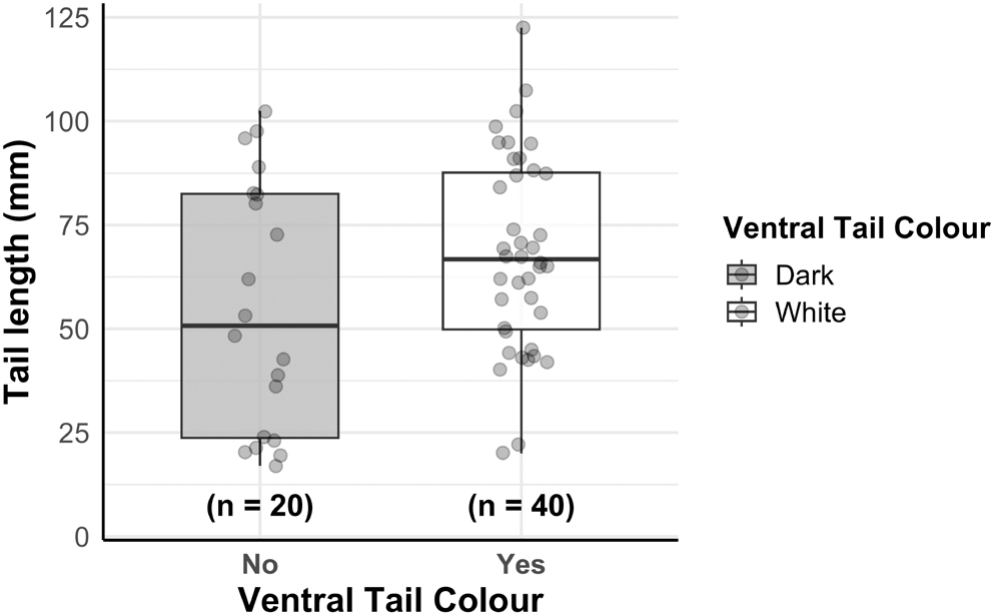
Boxplot showing the relationship between ventral tail colour and tail length (mm) across lagomorph species. Each dot represents a species, and the box shows the interquartile range, with the median marked by a horizontal line. Whiskers represent the minimum and maximum values. Sample sizes for each group are shown below the x‐axis.

If tail‐flagging behaviour is used as a signal for communication, then species with white tails should have a longer tail length than those with darker tails in order to maximise the effectiveness of the signal. Indeed, absolute tail length is longer for species with white tails than for those with darker tails (MCMCGLMM: *N* = 91, 95% CI [0.0150, 4.55], *p* = 0.036; Figures 5 and 6). Even if species without a tail (i.e., pika species) are removed from the model, the significant association is retained (MCMCGLMM: *N* = 62, 95% CI [0.326, 5.390], *p* = 0.014). Finally, group‐living species are more likely to evolve white tails (MCMCGLMM: *N* = 81, 95% CI [−0.249, 9.536], *p* = 0.038), which may suggest an intraspecific communication function.

### 3.2 Vigilance Advertisement

Overall, 8.4% of the European rabbits flagged their tail before seeing humans, the predator or control models. If rabbits tail‐flag to advertise their vigilance to potential undetected predators, then the probability of flagging should be higher in alert than non‐alert rabbits (Table 1). However, only 6.1% of alert rabbits flagged pre‐detection, which is even less likely than flagging by non‐alert rabbits (11.1%; GLMM: *β* = −2.010, SE = 1.038, *p* = 0.053).

### 3.3 Perception Advertisement

Since European rabbits might flag preflight towards conspecifics for alarm signalling purposes, only solitary rabbits were included in this analysis. If flagging preflight is to advertise perception, then individuals should be more likely to flag if they were approached by a fox than a marten (Table 1). However, after the predator had been seen, only 4/25 rabbits flagged when they were approached by a fox, and 2/31 for a marten (GLMM: *β* = −3.817, SE = 1.540, *p* > 0.1).

### 3.4 Quality Advertisement

Since flagging in flight may function as alarm signalling towards conspecifics, again only solitary European rabbits were included in this analysis to control for any effect of alarm signalling. If tail‐flagging in flight is to advertise quality, then flaggers should be running at a faster speed than non‐flaggers so as to honestly demonstrate physical fitness and probability of outrunning the coursing predator (Table 1). Indeed, during human approaches, flaggers ran at significantly faster speeds than non‐flaggers (average speed = 4.40 and 3.16 m/s, respectively; GLMM: *β* = 0.420, SE = 0.182, *p* = 0.02), but not against any other treatments (GLMM: *p* > 0.1 for all treatments).

Myxomatosis might also affect flagging in flight—healthier rabbits should be more likely to flag than those diseased (Table 1). A total of 18 solitary myxomatosis European rabbits were observed—nine with slight symptoms and nine with more severe symptoms. Overall, there was no difference in the running speed between rabbits with or without myxomatosis (LMM: *β* = 0.017, SE = 0.232, *p* > 0.1), although those diseased rabbits did escape at shorter FIDs than healthy rabbits (LMM: *β* = −1.01, SE = 0.256, *p* < 0.001). When approached by a human, rabbits with more severe symptoms of myxomatosis were significantly less likely to flag than those healthy individuals only (GLMM: *β* = −2.295, SE = 0.907, *p* = 0.011), but there was no difference in the proportion of flagging between healthy and slightly diseased rabbits (GLMM: *β* = −0.802, SE = 0.952, *p* > 0.1). When approached by a stuffed fox (*N* = 25) or stuffed rabbit (*N* = 19), tail‐flagging behaviour in flight did not vary with running speed or myxomatosis (GLMM: *p* > 0.1 for both treatments, respectively). No diseased rabbit was approached by the stuffed marten (*N* = 31), trolley (*N* = 20), or the live buzzard (*N* = 15).

### 3.5 Alarm Signalling

The stuffed European rabbit used in this study had a flagged tail, and if tail‐flagging is to alarm conspecifics, then the stuffed rabbit would not be treated as a control, but as a signal of potential danger instead. Thus, we removed the stuffed rabbit in this part of our analysis.

**FIGURE 7:**
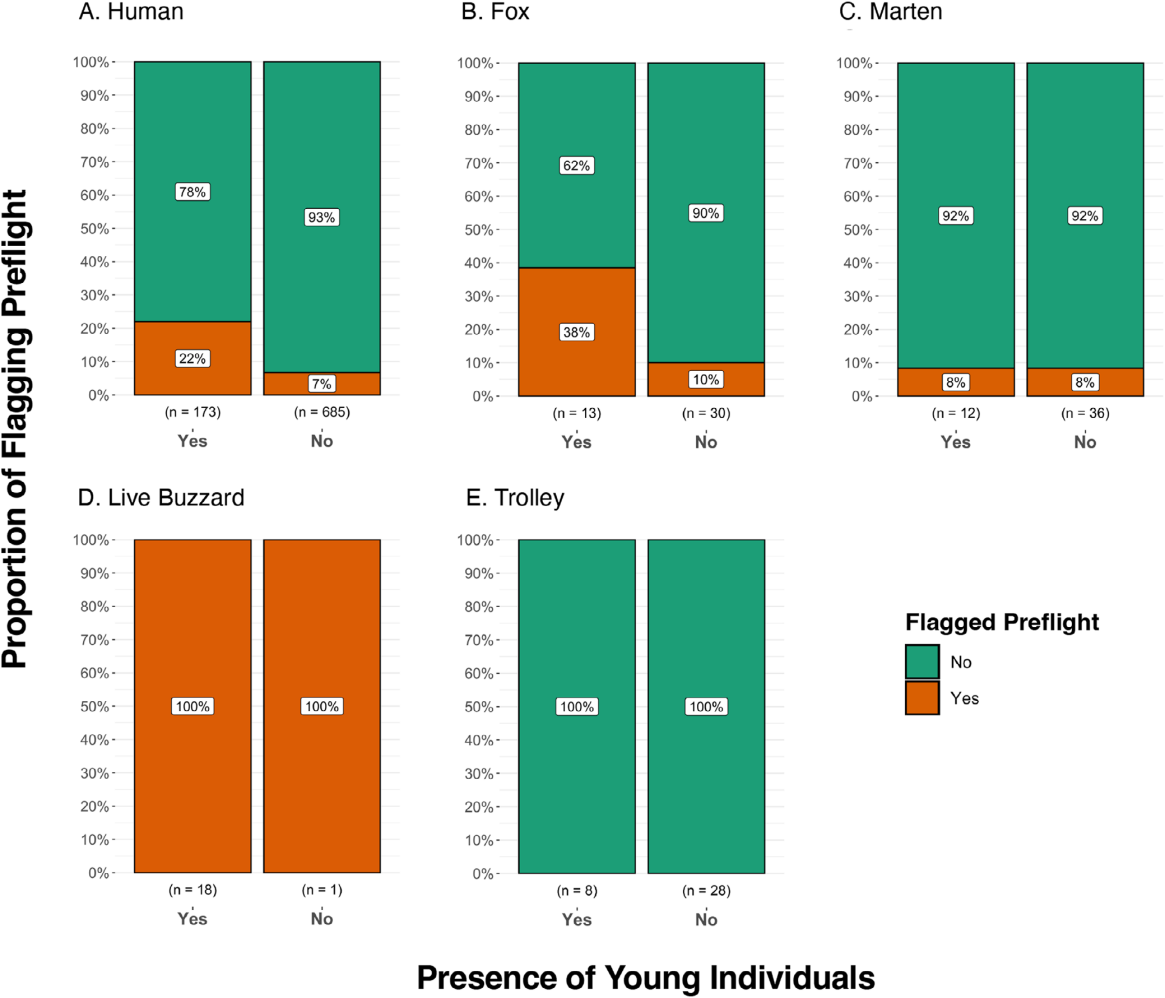
The proportion of tail‐flagging behaviour preflight for adult European rabbits with and without youngsters around. Each panel shows the types of approaching predators or controls. The sample size for each group is shown below the bar.

If tail‐flagging serves to warn a conspecific about potential danger, then the presence of offspring should increase the likelihood of flagging in adults both *preflight* and *inflight* through kin selection (Table 1). Before escape, adults were three times more likely to flag if they had young individuals around them compared to those adults without young when approached by humans (22% and 7%, respectively; GLMM: *β* = 1.263, SE = 0.391, *p* < 0.01; Figure 7A) and by the fox (38% and 10%, respectively; GLMM: *β* = 1.727, SE = 0.834, *p* = 0.038; Figure 7B). This difference was not observed when the adults were approached by a stuffed marten (8% for both adults with and without youngsters; GLMM: *β* = −4.571e‐8, SE = 1.206, *p* = 1; Figure 7C); all European rabbits flagged when approached by a live buzzard (Figure 7D); and no adults flagged when approached by the empty trolley (Figure 7E). Furthermore, adults were also more likely to flag preflight when approached by humans if there were a greater number of youngsters around them (GLMM: *β* = 0.179, SE = 0.092, *p* = 0.051). But this trend was not seen for the fox or any other treatments.

During the escape, adults with offspring present were still more likely to flag inflight when approached by humans (GLMM: *β* = 0.948, SE = 0.404, *p* = 0.019), but the likelihood of flagging inflight did not increase with more offspring escaping in the same group (GLMM: *β* = 0.250, SE = 0.406, *p* > 0.1). Similarly, there was no difference in the likelihood of flagging between adults with and without the presence of youngsters when they were approached by other threats (GLMM: *p* > 0.1 for all treatments).

Finally, across the phylogeny, there should be an association of white tails with larger group size and litter size, as those species should obtain a greater inclusive fitness if they flag their white tails (Table 1). As predicted, lagomorph species that live in larger group sizes were significantly more likely to possess white tails (MCMCGLMM: *N* = 81, 95% CI [0.07964, 8.25057], *p* = 0.042; Figure 5). Species that produce larger litter sizes were also marginally more likely to have white tails (MCMCGLMM: *N* = 63, 95% CI [−0.125, 2.615], *p* = 0.066; Figure 5).

### 3.6 Collective Motion

If collective motion is a function of tail‐flagging behaviour in flight, then solitarily running rabbits should not tail‐flag, and the proportion of rabbit tail‐flagging should be higher if they flee as a whole group (Table 1). Of those European rabbits that escaped alone, 83.6% of them flagged inflight (*N* = 1521, Figure 8). Thus, these solitary rabbits must be tail‐flagging for purposes other than maintaining group cohesion. During escape, 57% of the rabbit groups escaped as a whole group at the same time in the same direction, but they were not more likely to flag than those that did not escape as a group (GLMM: *β* = 0.234, SE = 1.102, *p* > 0.1).

**FIGURE 8:**
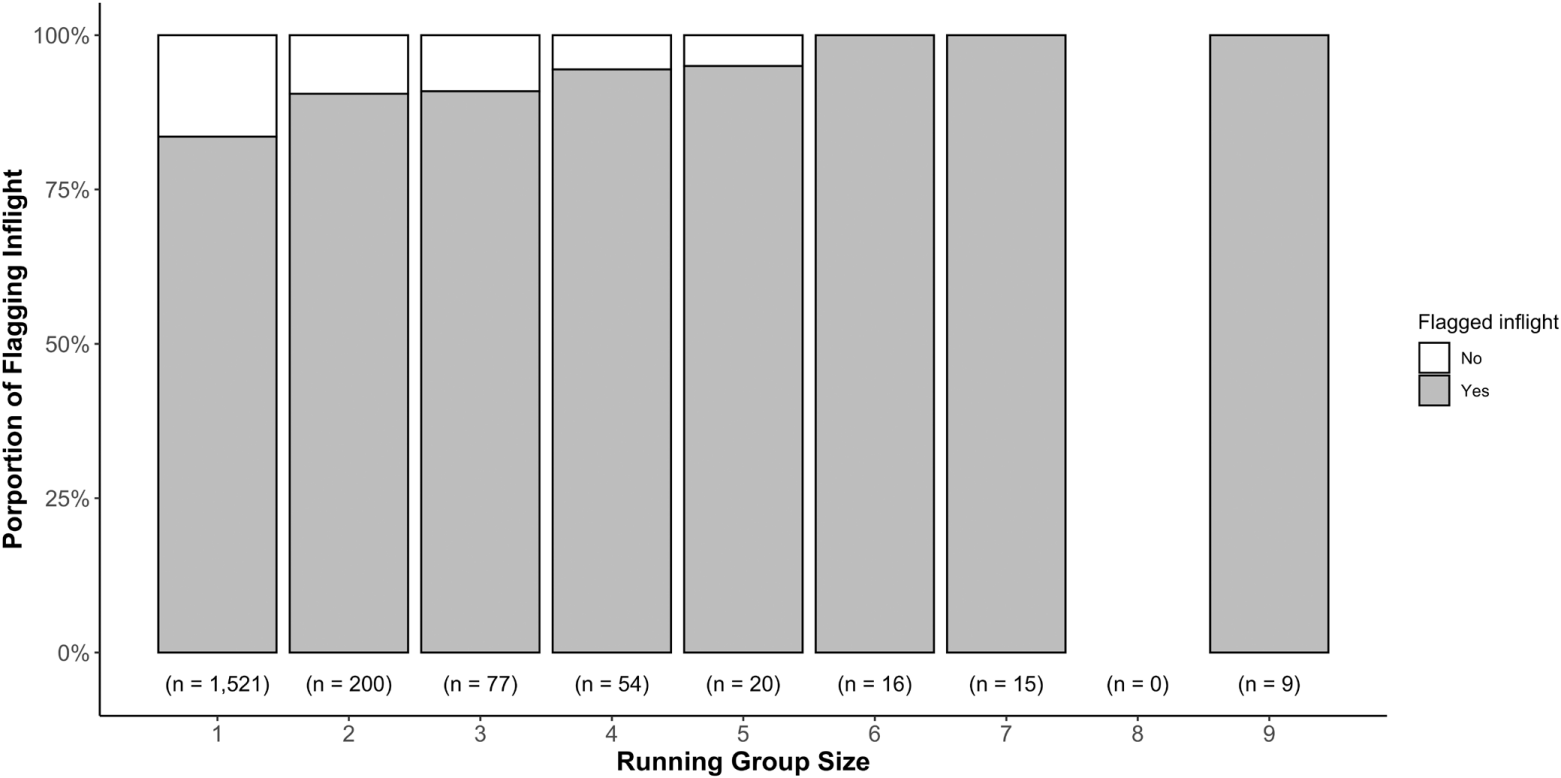
The proportion of individual European rabbits that tail‐flagged during the escape plotted against running group sizes across all treatments. Rabbits were grouped if they escaped together at the same time with other individuals from their initial group.

### 3.7 Confusion Effect

If rabbits flag in flight to promote group confusion, then all rabbits from the group should escape together, especially if approached by a predator (Table 1). Evidence for this was mixed: of those European rabbits found in groups, only 53.3% of them escaped altogether as a whole group when they were approached by a human. For the fox, 76.7% escaped as a whole group, 82.7% for the marten, 66.8% for the rabbit, 65.6% for the trolley and 100% for the buzzard. Thus, rabbits were more likely to escape as a whole group when they were approached by a live buzzard than a stuffed rabbit or an empty trolley (Tukey HSD, *p* = 0.003, and *p* < 0.001, respectively), which might lead to the group confusion effect when the group size is large enough (running group size of rabbits fleeing from buzzard: *N* = 14, mean = 6.79, SD = 3.14).

Another predictor for the group confusion effect would be that most rabbits escaping in a group should flag in flight (Table 1). Indeed, the likelihood of flagging inflight increased with the number of conspecifics that the European rabbits escaped with (GLMM: *β* = 0.437, SE = 0.136, *p* = 0.001; Figure 8). Rabbits were also more likely to tail‐flag inflight when they were approached by a fox or a human compared to a stuffed rabbit (Tukey HSD, *p* = 0.04 and *p* < 0.001, respectively).

### 3.8 Flash Behaviour

In flash behaviour, rabbits that flagged in flight should drop their tails post flight. In our study, a total of 373 solitary European rabbits tail‐flagged in flight—295 of them dropped their tails at the end of the flight, and 78 remained flagging (two‐sided binomial test, 95% CI [0.7460, 0.8310], *p* < 0.001). The FID and/or running distance should be longer for those rabbits that dropped their tails as finding the cryptic rabbits at the end would be harder if they were further away (Table 1). Indeed, individuals that dropped their tails also had a longer FID (GLMM: *β* = 0.421, SE = 0.202, *p* = 0.037) and a slightly longer running distance (GLMM: *β* = 0.396, SE = 0.229, *p* = 0.083) when approached by humans. However, we could not test any difference in the proportion of tail‐dropping postflight between other treatments due to the small sample size, as over 80% of rabbits ran into safety when they were approached by predators/controls other than a human.

Flash behaviour would work best for species with white tails and that are well camouflaged at rest (Table 1). This is because highly conspicuous prey would be too easy to find for flash behaviour to work (Sherratt and Loeffler‐Henry 2022). However, there was no association between the evolution of white tails and the lack of body conspicuousness across the phylogeny (MCMCGLMM: *N* = 87, 95% CI [7.947, 4.973], *p* = 0.630).

## 4 Discussion

Tail‐flagging behaviour in European rabbits likely serves two or more functions: as an alarm signal towards conspecifics before their escape and as a quality advertisement signal during flight in order to deter predator pursuit by some predators. At high‐population densities, flagging in flight could also contribute to a confusion effect, enhancing survival chances. Here, we discuss the relevant evidence supporting or contradicting each hypothesis highlighted in Table 1.

### 4.1 White Tails as a Signal

Across the lagomorph phylogeny, white tails are more likely to evolve in species with longer tail lengths, suggesting that white tails are used as a signal to convey information. European rabbits were more likely to tail‐flag preflight in less covered ground environments, suggesting they are signalling towards ground predators or conspecifics, but not aerial predators preflight. During the escape, tail‐flagging was more commonly seen under both less covered ground and aerial environments, supporting all potential signalling hypotheses. At the end of the escape, there was no association between flagging and environmental cover, suggesting either that the rabbits are not signalling towards any animals, or to hide for flash behaviour.

### 4.2 Vigilance Advertisement

Our results did not support vigilance advertisement as a potential function for the tail‐flagging behaviour pre‐detection in the European rabbits. A strong predictor for vigilance advertisement would be that alert rabbits were more likely to flag than non‐alert ones (Table 1); however, non‐alert rabbits were nearly twice as likely to flag pre‐detection compared to those who were alert. This finding contrasts with the typical expectation for vigilance signals, where increased alertness correlates with a higher probability of signal expression. For instance, in species such as white wagtails and ground squirrels, individuals exhibiting vigilant behaviours (e.g., scanning or reacting to previous predator encounters) are more likely to flag in anticipation of a threat (Randler 2006; Putman and Clark 2015). More generally, vigilance advertisement is an antipredator tactic that is poorly explored theoretically at present, and there are very few compelling studies to show that it is a widespread phenomenon in animals, in part because it involves separating vigilance in dangerous situations from that which would be expected from normal antipredator vigilance.

Those 8.4% of European rabbits that flagged pre‐detection could be signalling to conspecifics for communication outside of the predator–prey dynamic. For example, tail‐flagging may occur when males sniff females to determine the reproductive status of the female during the breeding season (Held et al. 2001). Rabbits may also flag their tail during social interactions, especially when young rabbits play and chase each other (Hawkins et al. 2008). Thus, the tail‐flagging behaviour before a predator has been seen may be addressed to conspecifics, rather than for signalling alertness to a potential predator, highlighting multiple uses of the same signalling trait.

### 4.3 Perception Advertisement

Similarly, our results did not support perception advertisement as a potential function for the tail‐flagging behaviour preflight in European rabbits. If perception advertisement is mainly targeted at stalking predators (Holley 1993; Ruxton et al. 2018a; Huang and Caro 2023), then we would expect a higher proportion of flagging preflight when the rabbits were approached by fox models (Table 1). Yet there was no difference between the proportion of individuals flagging when approached by a fox compared to a marten. In Holley's classic study, European hares only displayed standing behaviour at an intermediate distance from (live) foxes (Holley 1993), but in our study, the rabbits did not tail‐flag at an intermediate distance from the approaching stuffed fox. Instead, over 90% of rabbits escaped without flagging their tails before the flight at all.

Considering other lagomorphs more broadly, flagging is unlikely to operate as a perception advertisement signal in conjunction with the standing behaviour witnessed in brown hares, as they could not be seen simultaneously (i.e., standing would expose the white ventrum from the front, whereas the tail would be at the rear). Across mammals, the evidence for tail flagging as a perception advertisement remains equivocal. Tail‐flagging in white‐tailed deer ( *Odocoileus virginianus* ) was observed only after flight, while preflight flagging behaviour was subtle or absent (Caro et al. 1995). However, perception advertisement also could not operate later on in the flight, as the prey had already started escaping, so it would likely convey no extra information. Based on current evidence, we conclude that tail‐flagging behaviour preflight is not for perception advertisement.

### 4.4 Quality Advertisement

Our results provide some support for quality advertisement as a potential function for the tail‐flagging behaviour during the escape of European rabbits. An indicator of quality advertisement would be that rabbits with higher running speeds would be more likely to flag, perhaps indicating their physical ability to outrun a potential coursing predator and convincing the predator to abandon pursuit (Table 1). Indeed, we found that faster‐running solitary European rabbits were more likely to flag in flight. This pattern echoes findings in other species, such as the white‐tailed deer, where the flaggers ran faster than non‐flaggers (Caro et al. 1995) and in starlings ( *Alauda arvensis* ), where merlins ( *Falco columbarius* ) were less likely to catch singing individuals during pursuit (Cresswell 1994). Similar patterns have also been noted in ungulates, where those Thomson's gazelles that are in better physical conditions are more likely to engage in display behaviours like stotting (Fitzgibbon and Fanshawe 1988; T. Caro 1994), and in primates, where bright hindquarter markings have been linked to individual quality and signal salience during escape (Yu et al. 2022). Thus, conspicuous behaviours that may signal a prey's physical condition for the purposes of quality advertisement appear to be not uncommon across animal species.

Another moderately strong predictor for quality advertisement would be that healthy European rabbits should flag more than rabbits infected with myxomatosis (Table 1). This is because diseased rabbits may have a lower running speed and, thus, are less likely to escape pursuit. In our study, however, there was no difference in the running speeds of healthy or diseased rabbits. Nevertheless, severely diseased rabbits with myxomatosis were less likely to flag in flight than healthy rabbits. Rabbits with more severe symptoms have very swollen eyes, which may compromise their ability to physically see the approaching predator and, thus, result in their lower FID (Meredith 2013). If those rabbits could not identify the approaching object, whether it was a predator or a conspecific, then perhaps they were also less likely to flag in flight. This suggests that tail‐flagging under quality advertisement might not only signal running speed but also signal perceptual capacity.

Furthermore, European rabbits were more likely to flag inflight if they were at an intermediate distance from the nearest safety. This also supports quality advertisement as the rabbits would be unlikely to signal their ability to escape if they were very close to the predator and unlikely if they were so far away as to safely escape (Bergstrom and Lachmann 2001). Similar patterns have been observed in Thomson's gazelles, where individuals were more likely to stot at intermediate distances away from the predator (Fitzgibbon and Fanshawe 1988). In short, signalling quality may be most relevant at intermediate risk thresholds where predators' decisions can be influenced. Taken together, our results are suggestive but do not, as yet, provide robust support for quality advertisement as a function of tail‐flagging behaviour in European rabbits.

### 4.5 Alarm Signalling

We found strong support for alarm signalling as a potential function for the tail‐flagging behaviour in rabbits before, but not during, escape. If flagging serves to warn conspecifics about potential danger, then adults would be more likely to signal if probable offspring or kin were present (Table 1) and adult European rabbits with youngsters around them were three times more likely to flag when approached by a human or a fox model compared to adults without youngsters (Figure 7). Although we could not distinguish between males and females in this study, adults accompanying a youngster were likely females since males are not involved in parental care (DiVincenti and Rehrig 2016). This has been found in a wide variety of species, including ground squirrels and Siberian jays ( *Perisoreus infaustus* ), where females with offspring were more likely to produce alarm signals compared to those without offspring (Sherman 1977; Griesser and Ekman 2004).

This context‐specific signalling supports theoretical models suggesting that alarm signals, even if costly to the sender, can evolve through kin selection if they increase the survival of genetically related group members (Hamilton 1963; Piel 2016). In European rabbits, which live in social warrens and often rear young communally, such behaviour may be particularly adaptive. Similar strategies are also widespread in other mammalian and non‐mammalian taxa and are not just limited to visual signals. In mammals, for example, foot‐stamping in white‐tailed deer serves as a visual and auditory alert to group members (Caro et al. 1995) and tail‐flagging in fallow deer ( *Dama dama* ) may similarly function in intragroup communication, especially under open habitat conditions where such visual signals can travel efficiently (Alvarez et al. 1976; Caro et al. 2004). Among other vertebrates, many species such as minnows release chemical alarm cues (Pfeiffer 1977) and great tits produce predator‐specific alarm calls to alert conspecifics to predation (Suzuki et al. 2016). Insects also employ both chemical and acoustic alarms, such as the release of alarm pheromones in ants ( *Solenopsis invicta* ) and ultrasonic warning clicks in tiger moths ( *Bertholdia trigona* ) that function both as predator deterrents and conspecific alerts (Wilson and Regnier 1971; Corcoran et al. 2009).

As a note, none of the adults with youngsters flagged preflight when they were approached by an empty trolley (Figure 7E), lending indirect support to alarm signalling, as it could be a risk of being conspicuous to other potential predators and a waste of energy to signal to youngsters if no danger was detected. This cost–benefit trade‐off of signalling, also highlighted in studies on African bovids, reinforces the idea that honest signals are most likely to evolve and persist under conditions where the benefit to kin survival outweighs any increased individual risk (T. Caro 1994; Caro et al. 2004). Similar principles have been observed across taxa, where conspicuous warning or alarm displays are modulated based on predator presence, environmental context, or offspring proximity (Zahavi 1977; Maynard Smith and Harper 2003; Bradbury and Vehrencamp 2011). For example, in dampwood termites ( *Zootermopsis angusticollis* ), vibroacoustic alarm signals mobilise collective defence in genetically related colonies, despite risk to the signaller (Rosengaus et al. 1999).

Similarly, the alarm signalling function also predicts a relationship between signal evolution and group living. Across lagomorph species, we found that white tail presence was more common in species with larger social group sizes (Figure 5), consistent with interspecific predictions that conspicuous alarm signals are more likely to evolve in gregarious taxa (Caro et al. 2004). Furthermore, within the European rabbits, we also found that adults with larger litters were more likely to flag than those with fewer young, suggesting that alarm investment scales with potential fitness payoffs, similar to findings in vervet monkeys, where males with more offspring produce more alarm calls (Price et al. 2015).

Alarm signalling, however, was not supported as a potential function for the tail‐flagging behaviour *during* the escape. Having youngsters around the adult did not increase the likelihood of flagging in flight, which suggests that tail‐flagging in flight is not a signal directed at offspring. Adults with youngsters were also not more likely to flag if they were approached by a predator compared to an empty trolley. Rabbits may not need to alarm conspecifics during flight, as running is already a conspicuous signal sufficient to alert youngsters about potential approaching danger. Thus, we conclude that tail‐flagging behaviour preflight is highly likely to act as an alarm signal towards conspecifics and youngsters, but not in flight.

### 4.6 Collective Motion

Our results did not support group cohesion as a potential function for rabbit's tail‐flagging behaviour during flight. A strong predictor for collective motion would be that solitarily running individuals should not tail‐flag in flight as there are no conspecifics around to maintain group cohesion (Table 1). However, over 83% of solitarily running European rabbits escaped with a flagging tail in our study (Figure 8). This evidence directly challenges the fundamental expectation of collective motion, which requires conspecifics to stay together in a group (Bode et al. 2010; Negro et al. 2020). In contrast, numerous fish species exhibit highly coordinated group escape responses where visual cues, such as flashes or directional changes, help synchronise movement and enhance predator evasion. For instance, golden shiners ( *Notemigonus crysoleucas* ) form polarised schools in response to threats, using rapid information transfer to maintain cohesion and reduce individual predation risk (Ioannou, Singh, and Couzin 2012; Rosenthal et al. 2015). These examples highlight the contrast between taxa in how collective motion functions during escape.

Theoretical models and empirical evidence suggest that staying together during escape improves survival via dilution, confusion or selfish herd effects (Hamilton 1971; Hogan et al. 2016), and signals such as tail‐flagging could serve to maintain group cohesion while in motion (Ward et al. 2018; Yu et al. 2022). Indeed, conspicuous hindquarter markings have been proposed to aid in group alignment and spacing in taxa such as antelopes (Guthrie 1971; Yu et al. 2022), but these taxa often display tight spatial coordination in escape routes, unlike rabbits. However, our findings did not conform to these predictions. For instance, we found that 31/62 solitarily living lagomorph species evolved white tails (Figure 5), despite predictions that solitary species should not evolve such conspicuous traits if they primarily serve group cohesion (Table 1).

A moderately strong predictor for collective motion would be that the proportion of rabbit tail‐flagging in flight should be higher if they escape as a whole group in order to maintain group cohesion (Table 1). Although this pattern is common in taxa such as fish schools or bird flocks, where coordination during motion is key (e.g., Lagory 1981; Randler 2006), it was not reflected in our data. In our study, we found that many European rabbit groups scattered during the escape, and individuals did not manoeuvre to follow other group members. Furthermore, on average, only half of the escape events consisted of rabbits fleeing as a whole group and not leaving any group members behind. This is unlike the usual collective motion behaviour seen in other species, such as fish schooling or ungulate herding, where nearly all individuals actively follow the escape motions and route of group members (Lagory 1981; Herbert‐Read et al. 2015; Ward et al. 2018). Similar results were also found in white‐tailed deer, where solitary individuals tail‐flagged in flight and individuals did not bunch in response to flagging (Caro et al. 1995). It seems unlikely that tail‐flagging in rabbits acts as a signal to conspecifics to maintain cohesion during the escape.

### 4.7 Confusion Effect

In general, our results did not support group confusion as a potential function for the tail‐flagging behaviour during flight in European rabbits. If flagging in flight is used to confuse the predator, then a strong predictor would be that all rabbits from the group should escape together and most of them should flag in flight to ensure the effectiveness of the group confusion effect (Table 1). However, most European rabbits were found alone and escaped alone, with < 1% of escape events consisting of more than five rabbits running together in a group. Note that the group size used by many previous studies to demonstrate the confusion effect was all far larger than five individuals (e.g., three‐spined stickleback ( *Gasterosteus aculeatus* L.) attacking daphnia ( *Daphnia magna* ; Ioannou et al. 2008), largemouth bass ( *Micropterus salmoides* ) capturing silvery minnows (*Hybignathus nuchalis*; Landeau and Terborgh 1986) and the mealworms (larvae of *Tenebrio molitor* )) attacked by leopard geckos ( *Eublepharis macularius* ; Schradin 2001). As the running group size in our study is very low, it seems unlikely that the white tail would confuse predators through group confusion.

Although our results did not support signalling for a group confusion effect, we did find evidence that European rabbits were more likely to flag if they ran off with a conspecific (Figure 8) and were more likely to escape as a whole group when they were approached by the buzzard than other model predators or controls. This could lead to a coincidental group confusion effect (Table 1) in densely populated areas or during the breeding season. Such incidental confusion effects have been proposed in mammals such as colobus monkeys ( *Colobus vellerosus* ), which rely on multiple conspecifics moving simultaneously to reduce capture risk (Nokelainen et al. 2024). Rabbits could be in greater danger when approached by their native predators, such as a buzzard than by a human, and thus, were more likely to escape with conspecifics. Observations with real predation events are needed in future studies to confirm that predators are actually confused by tail‐flagging behaviour when rabbits do escape in large groups, a challenging request.

### 4.8 Flash Behaviour

Finally, our results are insufficient to conclude whether tail‐flagging inflight and dropping postflight work together to achieve flash behaviour in rabbits. Although nearly 80% of European rabbits dropped their tails at the end of the flight, which could indicate they are actively using flash display, it could also be a passive behaviour if they are no longer signalling for other functions, such as alarm warning. One supporting piece of evidence for flash display comes from the finishing position of those solitary rabbits that dropped their tails postflight: this was marginally further away from the predator compared to those that continued flagging after stopping. This might make finding the location of those individuals harder, as found in a previous study using human‐participated computer games (Loeffler‐Henry et al. 2021). However, there was no difference in the proportion of tail‐dropping postflight when rabbits were approached by a stuffed predator compared to a stuffed conspecific.

Flash displays are taxonomically widespread and have evolved in many cryptic species, such as grasshoppers, butterflies and birds, to mislead predators during escape (Cott 1940; Loeffler‐Henry et al. 2019). A prediction for flash behaviour from the comparative analysis would be that species with white tails should normally be well camouflaged (Bae et al. 2019; Table 1). However, there was no association between having white tails and overall body conspicuousness across lagomorphs. One explanation for this could be the difficulty in categorising conspicuousness across lagomorph species (Sherratt and Loeffler‐Henry 2022). Furthermore, in artiodactyls, the facultative exposure of conspicuous hindquarters is more common in solitary or small group‐living artiodactyl species, suggesting an interspecific signalling purpose potentially for flash behaviour (Caro et al. 2020). Yet, this was not again supported in lagomorphs as white tails were more likely to evolve in larger social group sizes (Figure 5). More direct field evidence related to the search time required to find the prey with tail dropping is needed, either with real predators or human‐participated computer games, looking at real‐life recordings of fleeing rabbits with or without a flagged tail at the end of the flight.

As with all field studies, our work includes certain constraints that may have influenced how European rabbits' responses were interpreted. First, we were unable to distinguish between male and female adult rabbits. Our sample size for adults without youngsters approached by a live buzzard was extremely limited (*N* = 1; Figure 7D) and should therefore be interpreted with caution. Furthermore, although martens are not native to our field sites, we used a taxidermy marten model due to the lack of available mustelid specimens and because similar musteline predators (e.g., stoats, weasels and polecats) are locally present. We note, however, that the model's head was mounted facing backwards, which may have altered how the rabbits perceived the predator's intent, potentially confounding the flight initiation response (see (Engelhard and Weladji 2011)). Although our findings provide important insights into tail‐flagging behaviour, we were not able to consider variation in predator visual systems. The assumption that the white tail is equally conspicuous to all predators may not hold, as foxes, for instance, are dichromatic, whereas avian predators such as buzzards are tetrachromatic and may perceive contrast differently (Kelber et al. 2003; Cuthill et al. 2017). Thus, what appears highly visible to human observers may not reflect predator perception; modelling conspicuousness of white fur at varying distances through the eyes of different predators would be beneficial (Nokelainen et al. 2024). Finally, additional explanations for the presence or absence of a white tail may relate to background matching or countershading (Caro et al. 2004), or to the physiological costs of melanogenesis (Ruxton et al. 2004). However, assessing these predictions falls outside the scope of this study, so this possibility remains speculative. Further work is needed to evaluate these alternative hypotheses.

## 5 Conclusion

In summary, our results indicate that raising the white tail has evolved for signalling in European rabbits but conveys different information at different escape stages from a predator. Exposing a conspicuous white tail probably informs relatives about potential danger before the escape. Later on, flagging a white tail could be used as a quality advertisement signal during escape to deter predator pursuit. If the rabbits are found in a densely populated area, then flagging during flight may coincidentally confuse a predator. We have ruled out vigilance and perception advertisement, and collective motion as a potential function for the tail‐flagging behaviour in lagomorphs, and there was insufficient evidence to conclude that flash behaviour is operating.

The extent to which these preliminary findings that tail‐flagging is an alarm signal to conspecifics and may inform the predator of the prey's ability to outrun can perhaps be extended to other closely related lagomorphs, which can facultatively expose their white hindquarters, at least as a set of working hypotheses. However, other functions may be involved in species in which white fur is always displayed like the white‐sided jackrabbit. Similarly, the function of white rumps in many artiodactyls that have white rumps always on display may differ as well (Caro et al. 2020). At present, it is premature to extrapolate from this study to other species without further research.

Nonetheless, multiple functions of a single antipredator defence may apply to these species (see Cooper 2001; Barbour and Clark 2012; Kikuchi et al. 2023). For example, the tail‐flagging behaviour in ground squirrels serves both as a vigilance and perception advertisement signal towards rattlesnakes. The former was demonstrated as the squirrel flagged more in areas previously associated with danger even when no predators had been detected yet (Putman and Clark 2015), and the latter was confirmed as the squirrels that flagged responded faster to an attack (Barbour and Clark 2012). Similarly, the same defensive morphology could also achieve multiple functions depending on context (Huang and Caro 2023). The orange‐black striping of the cinnabar moth caterpillar ( *Tyria jacobaeae* ) could be used as both a cryptic and aposematic colouration depending on the viewing context, such that it can first minimise predatory detection and also remind the predator of its unprofitability after being detected (Barnett and Cuthill 2014). Our knowledge of multiple functions of a single antipredator defence is increasing, and our study of tail‐flagging in European rabbits adds to this list and may well apply to other mammals that can facultatively expose conspicuous colour patches.

## Author Contributions

**Yuqian Huang:** data curation (lead), formal analysis (lead), funding acquisition (lead), methodology (lead), writing – original draft (lead), writing – review and editing (equal). **Reuben Evan Sparke:** data curation (supporting), writing – review and editing (equal). **Tim Caro:** conceptualization (lead), methodology (supporting), supervision (lead), validation (lead), writing – review and editing (equal).

## Conflicts of Interest

The authors declare no conflicts of interest.

## Supporting information


Appendix S1.

Appendix S2.

## Data Availability

The data that support the findings of this study can be found in the Appendix S1 and S2.
